# Crosstalk between the M_1_ muscarinic acetylcholine receptor and the endocannabinoid system: A relevance for Alzheimer's disease?

**DOI:** 10.1016/j.cellsig.2020.109545

**Published:** 2020-06

**Authors:** Karen J. Thompson, Andrew B. Tobin

**Affiliations:** Centre for Translational Pharmacology, Institute of Molecular Cell and Systems Biology, Davidson Building, University of Glasgow, Glasgow G12 8QQ, UK

**Keywords:** Alzheimer, Acetylcholine, Cannabinoid, Crosstalk, Cholinergic, Endocannabinoid, AD, Alzheimer's disease, mAChR, muscarinic acetylcholine receptor, Aβ, amyloid-beta, CNS, central nervous system, ACh, acetylcholine, AChE, acetylcholinesterase, nAChR, nicotinic acetylcholine receptor, GPCR, G protein coupled receptor, PLC, phospholipase C, MAPK, mitogen-activated protein kinase, PAM, positive allosteric modulator, NAGly, *N*-arachidonyl glycine, 2-AG, 2-arachidonoyl glycerol, AEA, *N*-arachidonoylethanolamine, DAG, diacylglycerol, LPI, lysophosphatidyl inositol, MAGL, monoacylglycerol lipase, FAAH, fatty acid amide hydrolase, PPARα, peroxisome proliferator-activated receptor α, Δ^9^-THC^1^THC, Δ^9^-tetrahydrocannabinol, CBD, cannabidiol, CHO, Chinese Hamster Ovary, NAM, negative allosteric modulator, ACEA, arachidonyl-2′-chloroethylamide, ACPA, arachidonylcyclopropylamide, PTX, pertussis toxin, CREB, cAMP response element binding, autLTD, autaptic long term depression, ROCK, RhoA/Rho-associated protein protein kinase, EGFP, enhanced green fluorescent protein, APP, amyloid precursor protein, PSEN1, presenilin-1, OP, organophosphorous

## Abstract

Alzheimer's disease (AD) is a neurodegenerative disorder which accounts for 60–70% of the 50 million worldwide cases of dementia and is characterised by cognitive impairments, many of which have long been associated with dysfunction of the cholinergic system. Although the M_1_ muscarinic acetylcholine receptor (mAChR) is considered a promising drug target for AD, ligands targeting this receptor have so far been unsuccessful in clinical trials. As modulatory receptors to cholinergic transmission, the endocannabinoid system may be a promising drug target to allow fine tuning of the cholinergic system. Furthermore, disease-related changes have been found in the endocannabinoid system during AD progression and indeed targeting the endocannabinoid system at specific disease stages alleviates cognitive symptoms in numerous mouse models of AD. Here we review the role of the endocannabinoid system in AD, and its crosstalk with mAChRs as a potential drug target for cholinergic dysfunction.

## 1 Introduction

50 million people worldwide currently live with dementia and, with an increasingly ageing population, this figure is expected to rise to >150 million worldwide by 2050, becoming the second leading cause of morbidity in the developed world after cancer [1]. The current estimated financial cost of dementia in the United Kingdom is £26.3 billion per year. Although the National Health Service and social services cover approximately £14.6 billion of this, some £17.4 billion – two thirds of the total cost – is covered by patients and their families [2]. Of the 50 million worldwide cases of dementia, 60–70% of these are cases of Alzheimer's disease (AD).

AD is predominantly associated with memory loss, but symptoms also include agitation, psychosis, depression, apathy, disinhibition, anxiety and sleep disorders [3]. The pathological hallmarks of Alzheimer's disease are brain atrophy and neuroinflammation, which are thought to be largely provoked by the deposition of amyloid-beta (Aβ) peptide into neuritic plaques and neurofibrillary tangles of tau protein [4,5] although the role of these is still not entirely understood. However, the cholinergic system has long been implicated in the pathophysiology of AD, as a plethora of cholinergic pathways serving roles in conscious awareness, attention and working memory have been consistently found to be damaged in the brains of those with advanced AD [6,7]. Furthermore, cholinergic transmission is reduced in several key brain regions in AD including the hippocampus, which is associated with memory formation [8,9]. This has resulted in the development of the ‘cholinergic hypothesis’, which postulates that a loss of cholinergic function in the central nervous system (CNS) significantly contributes to the cognitive decline associated with AD [10] and, as such, represents a druggable target for AD. Consequently, current AD treatment strategies focus on improving acetylcholine (ACh) availability. Indeed, currently available drugs for AD – acetylcholinesterase (AChE) inhibitors – alleviate symptoms by increasing ACh availability in the synaptic cleft of affected brain regions. By augmenting synaptic ACh concentration, AD symptoms can be relieved and the rate of cognitive decline can be slowed. Although improving ACh availability clearly shows improvements in AD symptoms, AChE inhibitors are nonselective in nature, which renders a myriad of dose-related adverse effects – such as gastrointestinal disturbances and bronchoconstriction [8]. Furthermore, only 30–40% of patients are responsive to AChE inhibitors [11]. Therefore, the clinical usefulness of AChE inhibitors is limited.

### 1.1 Acetylcholine receptors in Alzheimer's disease

Current research in AD drug discovery therefore strives to find alternative routes to target the dysregulation of cholinergic signalling. Both nicotinic and muscarinic acetylcholine receptors (nAChRs and mAChRs, respectively) have been implicated in AD. Among the fast-acting, channel-forming nAChRs, the α7 nAChR is of particular relevance to AD pathology, given its high expression levels in the hippocampus and physiological role in the enhancement of learning and memory [12,13]. Furthermore, Aβ has been shown to enrich in regions abundant in α7 nAChR and elevated levels of the α7 nAChR-Aβ complex are thought to disrupt normal cholinergic signalling, including that involved in synaptic plasticity, ultimately resulting in cognitive dysfunction [12,14,15]. Indeed, administration of α7 nAChR agonists AZD0328 and SSR180711 has shown improved cognition in rodents [16,17], and therefore the α7 nAChR undoubtedly represents a promising drug target for AD treatment. However, the role of α7 nAChR in AD lies beyond the scope of this review, and is more extensively discussed elsewhere.

The mAChRs – of which five subtypes (M_1_-M_5_) exist – regulate numerous functions in both the central nervous system and the periphery [18]. mAChRs belong to the Class A G protein coupled receptor (GPCR) superfamily, and are thus large, membrane-bound proteins consisting of 7 transmembrane domains. mAChRs contain an orthosteric binding site, to which endogenous acetylcholine binds, and intracellular regions where interactions with G proteins take place. Upon agonist binding, the recruitment of G proteins initiates downstream signalling cascades; M_1_, M_3_ and M_5_ mAChRs are coupled to Gα_q/11_ proteins, while M_2_ and M_4_ mAChRs are coupled to Gα_i/O_. The recruitment of Gα_q/11_ typically initiates coupling to phospholipase C (PLC), subsequently promoting the release of intracellular calcium ([Ca^2+^]_i_). The release of [Ca^2+^]_i_ is generally excitatory in nature; for example by facilitating the propagation of neuronal excitation through ion channel opening and the release of neurotransmitters from intracellular vesicles. Meanwhile, Gα_i_ recruitment is inhibitory in nature; agonist binding to M_2_ and M_4_ mAChRs results in the downregulation of adenylyl cyclase activity and reduced levels of cyclic adenosine monophosphate (cAMP), while decreasing the activity of voltage-activated Ca^2+^ channels, and increasing coupling to mitogen-activated protein kinase (MAPK) pathway [18,19].

Of the five mAChR subtypes, M_1_ mAChR is the most abundantly expressed in the brain [20]. Of particular note, M_1_ mAChR has long been implicated in learning and memory in the hippocampus. Recent studies have indicated that M_1_ mAChR expression and signalling is maintained in terminal AD patients [21] suggesting that drugs targeting this receptor subtype will be efficacious even in late disease. Not all studies however support this notion but rather have reported a downregulation of M1 mAChR expression of approximately 50% in AD [22]. Encouragingly, activation of this receptor can have pro-cognitive effects, as illustrated by xanomeline, an M_1_/M4-preferring agonist, which elicited significant cognitive and behavioural improvements in AD patients [23]. M_1_ mAChR is thus considered a promising target for the treatment of AD. However, despite such initially promising results, xanomeline ultimately failed in clinical trials due largely to off-target peripheral gastrointestinal and cardiovascular adverse effects thought to be mediated by M_2_ and M_3_ mAChR activation [24]. Indeed, the highly conserved nature of the orthosteric site across all 5 mAChR subtypes has made generating selective muscarinic ligands a challenge that has seriously hampered drug discovery efforts [25]. Efforts to generate selective muscarinic ligands have recently focused on allosteric compounds which target sites which are spatially distinct to the orthosteric binding sites. Allosteric molecules that enhance the activity of the natural ligand acetylcholine – so called positive allosteric modulators (PAMs) – have shown promise by improving both cognitive symptoms and survival in mouse cholinergic-deficit models [26] and in murine prion neurodegeneration [27].

Collectively these studies have provided evidence that the M1 mAChR is a promising molecular target that can not only improve deficits in learning and memory in neurodegenerative disease but also slow the disease progression. This conclusion has encouraged numerous drug discovery efforts that have resulted in clinical studies of both orthosteric [28] as well as allosteric/bitopic ligands [29] which have provided evidence of clinical efficacy, supporting the M1 mAChR as a target in AD.

## 2 The endocannabinoid system

The cannabinoid system encompasses endogenous cannabinoids (endocannabinoids), plant-derived cannabinoids (phytocannabinoids) and their target receptors, all of which are Class A GPCRs. Numerous synthetic cannabinoids are now available for research purposes (Table 1). Cannabinoids have received particular public attention in recent years, with the cannabis-based compound Sativex and the synthetic cannabinoid Nabilone having been legalised for medicinal purposes in the UK since late 2018. However, their prescription under the NHS remains restricted.

**Table 1:** Summary of key endocannabinoids, phytocannabinoids and synthetic cannabinoids and their activity at endocannabinoid receptors. The pharmacology of the numerous cannabinoids discovered and synthetic cannabinoids now available are extensively reviewed elsewhere (*e.g.* [67]).

	Cannabinoid	CB_1_ activity	CB_2_ activity	GPR55 activity
*Endogenous Cannabinoids*	2-arachidonoyl glycerol (2-AG)	Agonist	Agonist	Agonist
	*N*-arachidonoylethanolamine (anandamide; AEA)	Agonist	Agonist	Agonist
	Lysophosphatidyl inositol (LPI)			Agonist
	*N*-arachidonyl glycine (NAGly)			Agonist
*Phytocannabinoids*	Δ^9^-tetrahydrocannabinol (Δ^9^-THC^1^; THC)	Partial Agonist	Partial Agonist	Agonist
	Cannabidiol (CBD)	Negative Allosteric Modulator	Partial Agonist	Antagonist
*Synthetic Cannabinoids*	WIN55,212–2	Agonist	Agonist	
	Arachidonyl-2′-chloroethylamide (ACEA)	Agonist, potent and selective		
	Arachidonylcyclopropylamide (ACPA)	Agonist, potent and selective		
	SR141716a (Rimonabant)	Antagonist		Antagonist
	AM251	Inverse agonist		Agonist
	JWH-133		Agonist	
	JWH-015		Agonist	Agonist
	AM630		Antagonist	
	SR144528		Antagonist	
	ML184			Agonist
	ML193			Antagonist

It was initially believed that the endocannabinoid system only contained two target receptors – CB_1_ and CB_2_. However, the recently deorphanised GPR55 is now also considered a cannabinoid receptor, given that – in addition to its endogenous activation by lysophosphatidyl inositol (LPI) and *N*-arachidonyl glycine (NAGly) [30,31] – it is activated by a breadth of *endo*-, phyto- and synthetic cannabinoids [[32], [33], [34], [35], [36], [37]]. Interestingly, GPR55 has fairly low sequence homology with both CB_1_ (13.5%) and CB_2_ (14.4%) [36,38], and lacks the classical cannabinoid binding pocket expressed by CB_1_ and CB_2_ [39], meaning that it is somewhat of an atypical cannabinoid receptor. Collectively, the endogenous ligands, their receptors, and the enzymes which break them down comprise the endocannabinoid system.

### 2.1 Endogenous endocannabinoid ligands and their synthesis

The primary endocannabinoids are 2-arachidonoyl glycerol (2-AG) and *N*-arachidonoylethanolamine (anandamide; AEA), which both activate CB_1_, CB_2_ and GPR55 with similar efficacy and nanomolar potency [36]. Within the CNS, 2-AG is formed postsynaptically from phosphatidylinositol through the actions of PLC and diacylglycerol (DAG) lipase. Likewise, AEA is produced postsynaptically, but in this case is a downstream product of both phospholipase A2 and PLC [15]. Interestingly, LPI – the primary endogenous ligand for GPR55 – is thought to lie downstream of PLC and DAG formation, meaning that both LPI and 2-AG arise from the same second messenger pathway [32,40]. Therefore, the activation of cells *via* receptors coupled to PLC and PLA2 pathways results in the formation of endocannabinoids. Endocannabinoids are retrograde neurotransmitters; within the CNS, they are released from the postsynaptic neuron into the synaptic cleft and signal in a retrograde manner to endocannabinoid receptors expressed on presynaptic neurons. In this way, endocannabinoid receptors behave as modulators of presynaptic neurotransmitter release.

2-AG and AEA are broken down by monoacylglycerol lipase (MAGL) and fatty acid amide hydrolase (FAAH) respectively [41]. These breakdown enzymes, much like AChE as discussed above, may be exploitable as drug targets in diseases which may benefit from targeting strategies designed to upregulate the endocannabinoid system. This will be discussed in greater detail below.

### 2.2 Endogenous cannabimimetics

AEA is one of many endogenous *N*-acylethanolamine fatty acid amides which are produced concomitantly with AEA, and indeed dihomo-γ-lonolenic acid, mead acid, and adrenic acid are also capable of binding cannabinoid receptors [42,43]. However, the most abundantly produced *N*-acylethanolamines include palmitoylethanolamide and stearoylethanolamide, which do not bind cannabinoid receptors. Instead, a role for palmitoylethanolamide in anti-inflammatory, analgesic, anti-epileptic and neuroprotective functions is thought to be mediated at least in part by the peroxisome proliferator-activated receptor α (PPARα) [44,45]. Indeed, palmitoylethanolamide has been suggested as therapeutically useful across a breadth of pathologies, including neurodegeneration (reviewed in [46]). Meanwhile, *N*-stearoylethanolamide treatment prevented the loss of α7 nAChRs and the accumulation of pathogenic Aβ bound to α7 nAChRs in the brains of mice treated with bacterial lipopolysaccharide, which otherwise exhibit α7 nAChR loss and pathogenic Aβ binding to α7 nAChR accumulation [47]. Furthermore, *N*-stearoylethanolamide improved episodic memory in these mice, indicating treatment with *N*-stearoylethanolamide demonstrates a therapeutic potential for the prevention of cognitive dysfunction caused by neuroinflammation such as that seen in AD.

Many *N*-acylethanolamines concomitantly produced with AEA – including pamitoylethanolamide and *N*-stearoylethanolamide, as discussed above – are more abundant in most animal tissues than AEA. It has therefore been recently speculated that the release of such *N*-acylethanolamines could be the primary downstream products of the pathways producing AEA (reviewed in [48]). The potential therapeutic benefits of the *N*-acylethanolamines for neurodegenerative diseases, as briefly outlined above, are of importance when considering targeting the endocannabinoid system. However, more in-depth research is required in order to fully elucidate the druggable potential of the cannabimimetic *N*-acylethanolamines.

### 2.3 Phytocannabinoids

Phytocannabinoids have been used for recreational and therapeutic purposes for thousands of years [49], but have been of particular public interest in recent years given numerous emerging reports of the therapeutic potential in otherwise uncontrolled epilepsy, pain disorders, emesis and multiple sclerosis-related spasticity. To date, over 100 phytocannabinoids falling into eleven different chemical classes have been identified. All phytocannabinoid classes and their purported actions are reviewed extensively elsewhere [49,50]. Of importance, however, are the Δ^9^-tetrahydrocannabinol (Δ^9^-THC^1^; THC) and cannabidiol (CBD) classes, which are typically the two most abundant phytocannabinoids in *Cannabis sativa* respectively [50,51]. While THC is the main psychoactive component of cannabis, CBD retains the analgesic and anti-inflammatory properties of cannabis while being devoid of psychotropic effects.

THC is a partial agonist at both CB_1_ and CB_2_, with similar binding affinity at both receptors in AtT-20 [52] and Chinese Hamster Ovary (CHO) [53] cell lines. However, a modest reduction in THC potency for CB_2_ in comparison to CB_1_ was seen in the inhibition of forskolin-stimulated cAMP accumulation [52]. The characteristics of THC at the CB_1_ receptor have been paralleled to those of anandamide in terms of affinity, partial agonism and lower efficacy at CB_2_ *in vitro* [54]. Meanwhile, THC acts as a full agonist at GPR55, with nanomolar potency in a GTPγS assay [36].

In contrast to THC, CBD appears to act as a negative allosteric modulator (NAM) at the CB_1_ receptor. The affinity for CBD is reportedly very low, with a meta-analysis indicating a K_i_ of 3245 nM for CBD at the CB_1_ orthosteric site [55]. However, submicromolar concentrations of CBD were sufficient to inhibit internalisation of endogenously expressed CB_1_ in the ST*Hdh*^Q7/Q7^ cell culture model of striatal medium spiny projection neurons [56], suggesting that CBD is capable of affecting CB_1_ signalling at concentrations lower than those required for orthosteric binding. Indeed, the affinity of CBD to the allosteric binding site (K_B_) on CB_1_ has been found to be between 270 and 352 nM in HEK293 cells, and between 278 and 333 nM in ST*Hdh*^Q7/Q7^ cells [57]. A degree of functional selectivity was seen in ST*Hdh*^Q7/Q7^ (but not HEK293) cells, but importantly CBD reduced the potency and efficacy of both THC and 2-AG in both of these cell lines [57,51]. Meanwhile at the CB_2_ receptor, CBD is an orthosteric partial agonist, capable of competing with the synthetic CB_2_ receptor agonist SR144528 [51]. Finally, CBD is a competitive antagonist of GPR55 [36,51], and is also capable *in vitro* of anandamide uptake inhibition, thus increasing endocannabinoid availability [58,59]. Indeed, this may well explain some of the reported therapeutic effects of CBD, given that the uptake mechanisms of anandamide and 2-AG are potential therapeutic targets, as discussed in detail below.

In addition to their activity at their putative endocannabinoid receptors, both THC and CBD modulate the activity of numerous additional cellular effectors. For example, THC has been found to inhibit 5HT_3A_-induced currents in HEK293 cells and cultured rat trigeminal ganglion neurons [60,61], while potentiating positive allosteric modulatory effects at TRPA1 [62] and TRPV2 [63]. Meanwhile, CBD also modulates 5HT_1A_ [64], GPR55 [36], μ- and δ-opioid receptors [65], TRPV1 channels [58], and PPARγ [66]. Therefore, when considering the therapeutic potential of cannabinoids, such off-target activity must be borne in mind. Given that the proportion of each chemical class – dependent on numerous aspects such as the strain of the plant, growing conditions, and processing methods – varies enormously, this can produce substantial pharmacological variations in *Cannabis sativa*. It is thus crucial to fully understand the pharmacological properties of each of the phytocannabinoids to exploit their potential, and the potential of the endocannabinoid system as a druggable target.

### 2.4 Synthetic cannabinoids

The discovery of the endocannabinoid system was soon followed by the production of numerous synthetic cannabinoid compounds in a bid to advance research and understanding around the endocannabinoid system and its potential for therapeutic exploitation. A plethora of such synthetic compounds now exists, and are extensively reviewed elsewhere (*e.g.* [67]). A summary of key synthetic compounds now widely used in research is provided in Table 1.

The highly potent, yet nonselective, CB agonist WIN55,212-2 (WIN55212) was first synthesised in 1992 [68]. This analogue of pravadoline exerts full agonism at both the CB_1_ [52,53,69] and CB_2_ receptors [70]. However, it is noteworthy that WIN55212 has higher affinity and efficacy at CB_2_ than CB_1_ [52] and, importantly, has neither binding nor functional activity at GPR55 [36].

Arachidonyl-2′-chloroethylamide (ACEA) and arachidonylcyclopropylamide (ACPA), first synthesised in 1999, are both analogues of AEA and are potent, selective agonists of the CB_1_ receptor [71]. Both of these compounds bind CB_1_ receptors with higher affinity than AEA, and inhibit forskolin-stimulated adenylyl cyclase activity in cells expressing the CB_1_ receptor, but not in cells expressing the CB_2_ receptor [71]. Rimonabant – SR141716a – is a selective antagonist for CB_1_ receptors, able to antagonise the effects of both anandamide and WIN55212, and is thus a competitive antagonist at the CB_1_ receptor [52,72]. Rimonabant was approved as an antiobesity drug in the EU in 2006, but was withdrawn from use in the EU in 2008 due to serious psychiatric adverse effects, including suicide. However, SR141716a remains widely used for research, along with the structurally similar AM251 which acts as a potent inverse agonist at CB_1_ receptors, to allow dissection of CB_1_ and CB_2_ receptor activity.

First synthesised in 1999, JWH-133 is a THC analogue which is a highly selective, full agonist for CB_2_ [73]. Excitingly, this synthetic cannabinoid has been shown to reduce spasticity in a murine model of multiple sclerosis [74], thus highlighting the therapeutic potential of selective synthetic cannabinoids. JWH-015 is also a selective CB_2_ agonist which is widely used to date for CB_2_ research [75,53]. In terms of antagonists, both AM630 and SR144528 are more potent at blocking CB_2_ activity over CB_1_ activity, exhibit higher affinity for CB_2_, and block agonist-induced CB_2_ activation in a competitive manner [67].

The activity of synthetic cannabinoids at GPR55 has yielded some surprising results. Both the CB_2_ agonist JWH015 and the CB_1_ antagonist AM251 evoke an increase in intracellular calcium in HEK293 cells expressing GPR55 [76]. Meanwhile, GPR55 is unresponsive to the nonselective CB agonist WIN55212, yet the CB_1_ antagonist SR141716a is also an antagonist at GPR55 [76]. Lastly, some selective synthetic cannabinoids now exist for GPR55 – ML184 is a potent and selective agonist at this receptor [34], while ML193 is a potent and selective antagonist [77].

## 3 CB_1_ and CB_2_ Receptors – the classical cannabinoid receptors

The CB_1_ receptor, first cloned in 1990 [78], are widely expressed in the central nervous system within both neurons and glial cells, where they are predominantly expressed at the terminals of neurons. Here, they serve as modulators of both excitatory and inhibitory neurotransmission, and mediate numerous brain functions including cognition [79]. Furthermore, CB_1_ activity protects against excitotoxicity and promotes repair mechanisms in response to neuronal damage [41].

Cloning of the CB_2_ receptor followed soon after in 1993 [70]. Its expression is classically associated with the periphery where it is largely implicated in immune functions [80], and is responsible for the anti-inflammatory effects of cannabis [81]. In peripheral immune cells, such as B lymphocytes, natural killer cells, monocytes, macrophages, microglia and T lymphocytes [80], CB_2_ is thought to modulate cell migration and cytokine release. CB_2_ is also found on microglial cells – the resident immune cells of the brain [82] – yet is barely detectable in the healthy brain [83]. However, evidence for neuronal CB_2_ expression in the hippocampus, and a resulting role in synaptic plasticity, has recently been described [81]. Furthermore, CB_2_ levels in the brain have been shown to be elevated in response to neuroinflammation and CNS damage, indicating a far more prominent role for CB_2_ in the CNS than originally believed (see discussion below).

Both CB_1_ and CB_2_ signal predominantly through Gα_i_/_o_ proteins, and thus in a manner similar to M_2_ and M_4_ mAChRs, inhibit adenylyl cyclase but have also been reported to increase activation of the MAPK pathway [41], inhibit voltage-dependent Ca^2+^ channels, and activate K^+^ channels [41]. Therefore, the predominant role of endocannabinoid receptors is to modulate cell excitability.

### 3.1 CB_1_ and CB_2_ are promiscuous GPCRs capable of oligomerisation

Although the majority of work on CB_1_ and CB_2_ receptors has focussed on their coupling to Gα_i/o_, both of these endocannabinoid receptors are now understood to be among the GPCRs capable of coupling to numerous G proteins in a manner often described as promiscuous coupling. For example, CB_1_ is proposed to heteromerise with the dopamine receptor type 2 long form (D_2L_), the μ-, κ-, and δ-opioid receptors, the orexin-1 receptor, the A_2A_ adenosine receptor (A_2A_) and the β_2_ adrenergic receptor (β_2_-AR) [[84], [85], [86], [87], [88], [89], [90]]. Importantly, CB receptor coupling to alternative pathways appears to be particularly prevalent in the case of oligomer formation [91]. Such alternative pathways can also be unmasked with the blockade of the Gα_i_ pathway with pertussis toxin (PTX), or can be dependent on the CB_1_ agonist used [91].

The most widely documented incidence of CB receptor promiscuity is CB_1_ coupling to Gα_s_ proteins [[91], [92], [93]], by which adenylyl cyclase is activated and cAMP response element binding (CREB) protein becomes phosphorylated [94]. As activation of GPCRs coupled to Gα_i/o_ – such as CB_1_ and CB_2_ receptors – results in the inhibition of adenylyl cyclase and a subsequent decrease in cAMP levels, the activation of GPCRs coupled to Gα_s_ therefore initiates an almost opposite cellular response to that which is typically associated with CB_1_ and CB_2_ receptors. The Gα_s_ phenotype was first seen upon co-stimulation of primary rat striatal neuronal cultures, and upon Gα_i/o_ inhibition in CHO cells expressing human CB_1_ (CHO-hCB_1_) [92]. When stimulated with either quinpirole (a D_2_ receptor agonist) or HU210 (a CB_1_ receptor agonist) alone, forskolin-induced cAMP was inhibited in striatal neurons in a typical Gα_i/o_-mediated response. However, co-stimulation with both quinpirole and HU210 reversed this inhibitory response, resulting in an augmented cAMP accumulation in a dose-dependent manner, indicating that CB_1_-D_2_ oligomers signal through Gα_s_ [92]. Meanwhile, this same study demonstrated that stimulation of CHO-hCB_1_ cells with HU210 resulted in a dose-dependent cAMP inhibition, yet blockade of Gα_i/o_ with PTX resulted in a dose-dependent cAMP accumulation in the absence of either quinpirole co-stimulation or indeed the D_2_ receptor, thus indicating that the CB_1_ receptor alone is sufficient to induce a non-Gα_i/o_ signalling profile [92]. A more recent study has indicated that combined D_2_ antagonism with haloperidol and CB_1_ agonism with ACEA in CB_1_-D_2L_ heteromers in ST*Hdh*^Q7/Q7^ not only enhances CB_1_ coupling to Gα_s_, but haloperidol in fact behaves as an allosteric modulator of CB_1_ coupling the Gα_s_ [95]. It has been hypothesised that the physiological relevance of such a functional switch is to provide discrete regulation of neurotransmitter signals [92].

Coupling of the CB_1_ receptor to Gα_q_ [96,97] has also been reported, with a physiological role for Gα_q_-coupled CB_1_ having been suggested in the induction of autaptic long term depression (autLTD). In primary hippocampal neuronal cultures, autLTD can be induced with an intermittent low-frequency stimulus. However, autLTD was strongly diminished when 2-AG production was blocked with a DAG lipase inhibitor, reversed when neurons were treated with the CB_1_-selective antagonist SR141716, and absent altogether in primary hippocampal neuronal cultures from CB1^−^/^−^ mice [97]. However, autLTD induction could not be prevented with the treatment of pertussis toxin and thus blockade of G_i/o_ [97]. Indeed, the induction of LTD is dependent on the presynaptic accumulation of PLC and filled Ca^2+^ stores, consistent with Gα_q_ coupling. While autLTD remains poorly understood in relation to other forms of synaptic plasticity and depression, this study also indicated that autLTD was dependent on either ionotropic or Group I metabotropic glutamate receptors. The authors thus concluded that the activation of the CB_1_ receptor and subsequent coupling to Gα_q_ confers the release of presynaptic glutamate [97].

Lastly, CB_1_ coupling to Gα_z_ has been implicated in cross-tolerance with the μ-opioid receptor arising from receptor desensitisation by regulation of adenylyl cyclase activity and the gating of certain K^+^ channels [98]. The CB_1_ receptor co-precipitated with histidine triad nucleotide binding protein 1 (HINT1) – a signalling motif associated with Gα_z_ coupling in the μ-opioid receptor – in the periaqueductal grey matter of mice. CB_1_ receptor association with HINT1 was further increased following WIN55212, although in mice where Gα_z_ protein levels had been decreased with oligodeoxynucleotides directed against Gα_z_, no WIN55212 desensitisation was seen [98]. Increased association with HINT1 was not seen following THC administration, indicating that only exposure to certain cannabinoids results in increased coupling to Gα_z_ and thus desensitisation [98]. The authors of this study therefore hypothesised that certain cannabinoids may determine a G protein bias upon ligand binding, a phenomenon which could therefore be of therapeutic use, as the choice of ligand could dictate G protein coupling and thus functional outcome.

CB receptor G protein promiscuity has also been observed in CB_2_ receptor coupling to Gα_s_ [99,100], which has recently been implicated in wound healing in the cornea [101]. However, there is a surprising paucity of data on this matter relative to that surrounding G protein promiscuity at CB_1_, indicating the need for further research in this field.

Both the cannabinoid agonist used and the expression level of Gα_i_ have been recently shown as crucial to the switch in G protein coupling in cannabinoid receptors [91]. Indeed, the functional switch from Gα_i_ to Gα_s_ coupling was reversible by both pharmacological knockdown with an irreversible CB_1_ antagonist (AM6544) and by increasing Gα_i_ expression levels [91]. Collectively, this has led to the hypothesis that alternative signalling pathways are initiated upon Gα_i_ exhaustion, due either to Gα_i_ inactivation or sequestration by another receptor such as the D_2_ dopamine receptor [91,95]. The dependency of G protein coupling on the choice of ligand indicates that, akin to many GPCRs, pathway bias in CB receptors may be therapeutically useful when targeting specific, disease-related pathways. The phenomenon of CB receptor promiscuity raises the possibility of the activation of different pathways in different physiological contexts, and highlights a far greater level of complexity in endocannabinoid signalling than was originally realised when CB_1_ and CB_2_ were first cloned in the early 1990s.

### 3.2 GPR55 – a recently deorphanised, third cannabinoid receptor

GPR55 has functions in both the periphery and the CNS. In the periphery, GPR55 is expressed in the adrenal glands, endothelial cells and the gastrointestinal (GI) tract [36,76,102]. Surprisingly, evidence has been found for both pro- and anti-inflammatory effects of GPR55 in the periphery. LPI produced by macrophages is increased during inflammatory conditions [103], while LPI and AM251 (an agonist at GPR55) induce a directional migration of human peripheral blood neutrophils [104], and activation of GPR55 increases pro-inflammatory cytokine release from monocytes and natural killer cells [[105], [106], [107]]. On the other hand, GPR55 has also been found to attenuate neuroinflammation and chronic pain in a dinitrobenzenesulfonic acid-induced colitis model [108,109]. It is therefore likely that the role of GPR55 is dependent upon physiological context and inflammatory conditions.

In the CNS, GPR55 is found in several regions of the brain, the caudate nucleus, putamen, hippocampus, thalamus, pons, cerebellum, frontal cortex and hypothalamus [36,102]. GPR55 has also been reported to be located on neural stem cells, where it is proposed to have functions in neural stem cell proliferation, neuronal survival, neuronal differentiation [30] and the regulation of growth cone morphology [110]. Of note, recent evidence indicates that GPR55 activation in the hippocampus can boost glutamate release and modulate synaptic plasticity in the CA1 region [111,112].

In contrast to CB_1_ and CB_2_ receptors, GPR55 is coupled to Gα_12/13_ [32,36,113] and Gα_q_ proteins [76,[114], [115], [116], [117]]. Activation of GPR55 therefore results in the activation of RhoA/Rho-associated protein protein kinase (ROCK), protein kinase B (Akt), and PLC pathways thus increasing neuronal excitability [33,76,118,119]. Much like the promiscuity of CB_1_, it is believed that GPR55 G-protein coupling is conferred by the ligand of choice [33]. It is still unclear which G protein is preferred by GPR55; both mice doubly deficient in Gα_q_ and Gα_11_ in nociceptive neurons (SNS-Gα_q/11_^−^/^−^) and those deficient in Gα_13_ (SNS-Gα_13_^−^/^−^) in nociceptive neurons showed a partial but significant reduction in LPI-induced hypersensitivity, indicating that both Gα_q/11_ and Gα_13_ contribute to LPI-induced hypersensitivity in the same physiological context *i.e.* nociceptive neurons of the DRG [120]. However, it remains plausible that, akin to CB promiscuity, G protein coupling of GPR55 could be dependent on physiological context. Indeed, much early work on GPR55 was carried out in HEK293 cells [32,36,76]; this therefore limits the conclusions which can be drawn about preferential G protein coupling *in vivo*.

## 4 The endocannabinoid system in Alzheimer's disease

The endocannabinoid system is one of many neurotransmitter systems affected by Alzheimer's disease. Alterations in CB_1_ receptor expression and functionality have been described in both AD animal models [121,122] and the brains of AD patients [[123], [124], [125]]. Interestingly, these alterations vary throughout the progression of AD. In the early stages of disease, both human AD patients and AD animal models exhibit an increase in CB_1_ activity and expression [122,125], perhaps as a neuroprotective response [126]. In contrast, late-stage AD is associated with a reduction of CB_1_ expression [123] in numerous brain regions including the CA3 and CA1 regions of the hippocampus [127,129].

Postmortem brains of AD patients have indicated that levels of MAGL and FAAH – the breakdown enzymes for 2-AG and anadamide, respectively – are upregulated [131]. Therefore, the early-stage increase in CB_1_ expression may be in part attributed to a concomitant upregulation of these enzymes and thus overall reduction in endocannabinoid availability. Upregulation of 2-AG *via* genetic deletion of MAGL in mice surprisingly enhanced LTP, object recognition and spatial memory, with a significantly decreased CB_1_ receptor binding density [128]. Pan et al. proposed that genetically enhanced 2-AG levels resulted in tonic activation and consequential desensitisation of the CB_1_ receptor [128]. It is thus plausible that AD-related upregulation of MAGL – initiating a downregulation of 2-AG availability – results in a compensatory upregulation of CB_1_ receptors and is therefore responsible for the increase in CB_1_ receptor density seen in the initial stages of AD.

Although CB_2_ receptor expression levels are relatively low in the healthy brain [83], expression of this receptor is upregulated in amyloid-associated neuroinflammation, seen in both human AD patients and rats inoculated with β-amyloid [123]. This has been illustrated in mice expressing five familial Alzheimer's disease mutations (5xFAD mouse model) with enhanced green fluorescent protein (EGFP) tagged CB_2_ receptor, which showed enhanced EGFP – and thus CB_2_R – expression at 3 months [129]. This 3 month point represents a point which precedes the onset of cognitive symptoms and deterioration of synaptic transmission in this mouse model, evident at around 6 months. EGFP-CB_2_R labelling was detected specifically in areas of intense inflammation and amyloid deposition and was coincident with the appearance of plaques in the cortex, hippocampus, brain stem and thalamus [129]. Furthermore, EGFP expression was restricted to activated microglial cells surrounding neuritic plaques, and was remarkably absent in non-plaque regions, indicating that the upregulation of CB_2_R is directly related to plaque development. This is consistent with observations from postmortem human tissue from patients with neurodegenerative disorders accompanied by inflammation, including AD [129]. It is not yet clear whether the upregulation of CB_2_ is a cause or effect of neuritic plaque formation, although it has been suggested that CB_2_ is induced under neuroinflammatory conditions [129]. Furthermore, given that the first incidence of CB_2_R upregulation in the 5xFAD mouse model correlates with amyloid deposit at 3 months, Lopez et al. [129] hypothesised that the induction of CB_2_ receptor expression in the CNS takes place after a period of sustained neuroinflammation, and even postulated that CB_2_ receptor expression in the CNS could be an early indicator of AD [129]. Given that CB_2_ receptor upregulation in the CNS precedes phenotypic symptoms of AD, this receptor could represent an early target for AD [129].

The endocannabinoid system is thus an attractive target system in AD, particularly in the early stages. Given that many AD symptoms arise from cholinergic dysfunction, and are paralleled with changes in the endocannabinoid system, there is therefore potential for crosstalk between the endocannabinoid and muscarinic systems. In particular, disease-related changes in endocannabinoid function may offer therapeutic possibilities in AD.

## 5 Crosstalk between muscarinic and endocannabinoid receptors – implications for Alzheimer's disease

Crosstalk has been described between both the nAChRs and mAChRs with the endocannabinoid system. Although α7 nAChR agonism with PNU282987 has been shown to be unaffected by both CB_1_ antagonism with rimonabant and CB_2_ antagonism with SR144528 [130], the α5 and α6 nAChRs have been recently implicated in THC dependency and withdrawal [131]. Therefore, although nAChR crosstalk with the endocannabinoid system may not be directly related to AD, this highlights the potential for the cannabinoid system to influence global cholinergic signalling and is thus a crucial consideration in the use of cannabinoids to target the cholinergic system in AD.

The crosstalk between CB_1_ and muscarinic receptors in AD has been recently investigated using the triple transgenic mouse model of AD – the 3xTg-AD model – in which mice are homozygous for the *PSEN1 (presenilin-1)* mutation, APP (amyloid precursor protein) Swe transgene, and tauP301L transgene. 3xTg-AD mice exhibit impaired synaptic plasticity, which is thought to arise from the dysregulation of hippocampal presynaptic muscarinic neurotransmission, thus inhibiting memory creation and maintenance [132]. In these mice, intraneuronal Aβ appears at 2–4 months alongside impaired mAChR-mediated signalling [133], with cognitive impairment being accompanied by elevated CB_1_ expression at around 7 months [126,134]. While cognitive impairment becomes more evident in middle-aged (13–15 months) mice, a simultaneous decrease in choline acetyltransferase and CB_1_ expression is seen [126,134]. In late life (18–20 months), these mice exhibit hippocampal and cortical cholinergic neuritic dystrophy, paralleled with the progression of amyloid-α plaque formation [132]. These changes in both mAChR and CB_1_ expression and function are parallel to those seen in human AD as discussed above. Indeed, a positive and statistically significant correlation was found between CB_1_ receptor density in the basolateral amygdala and acquisition latencies in 3xTg-AD mice, indicating that changes in CB_1_ density may contribute to deficits in acquisition [132]. In the 3xTg-AD mouse model, therefore, CB_1_ expression may influence cognitive function.

The elevated CB_1_ expression observed in 7-month-old 3xTg-AD mice may represent a therapeutic window. By stimulating the CB_1_ receptor with the synthetic agonist WIN55212, disease-associated acquisition latencies were reversed at this time point in 3xTg-AD mice to levels comparable with non-Tg control mice [132]. Furthermore, WIN55212 reversed disease-associated impairments in functional coupling of M_2_/M_4_ mAChRs to G_i/o_ proteins in the basolateral amygdala, lateral amygdala and hippocampal CA1 region [132]. Therefore, by both directly activating the CB_1_ receptor through agonist administration, Llorento-Ovejero et al. [132] reversed both cognitive and functional cholinergic deficits in 3xTg-AD mice. In the 5xFAD model, mice overexpress mutant human APP(695), while also expressing the Swedish, Floridian and London Familial AD mutations, in addition to two FAD mutations M146L and L286V, both of which become overexpressed in the brain due to their regulation by the mouse *Thy1* promoter. In contrast to the 3xTg-AD model, blockade of CB_1_ with SR141716 – a CB_1_-specific antagonist – exacerbated neuroinflammation in the 5xFAD mouse model [135]. Despite exacerbating neuroinflammation, antagonist treatment of the CB_1_ receptor neither attenuated nor exacerbated spatial memory in 5xFAD mice [135]. Meanwhile, the genetic deletion of CB_1_ has been shown to create an imbalance of excitatory/inhibitory neurotransmission in the hippocampus, drastically accelerating memory impairment and reducing survival in APP/PS1 transgenic mice. The APP/PS1 mouse model is a double transgenic mouse model, expressing a chimeric mouse/human amyloid precursor protein, and a mutant human presenilin 1(PS1-dE9), and represents a model of familial AD with cognitive impairments, Aβ plaque deposition and synaptic abnormalities from 6 months of age [138]. The effects of activating, blocking, or deleting the CB_1_ receptor in mouse models of AD therefore highlight the importance of CB_1_ in the modulation of AD-like symptoms, particularly those attributed to cholinergic dysfunction. Importantly, activation of the CB_1_ receptor in vulnerable limbic areas *via* a CB receptor agonist is capable of reversing symptoms of cholinergic deficit and reversing functional mAChR impairments, while blockade or genetic deletion of the CB_1_ receptor can exacerbate disease. Collectively, this indicates that the early upregulation of CB_1_ expression in AD may serve a pivotal, and druggable, role in an attempt to protect cholinergic function *via* CB1-mediated pathways.

While direct activation of CB_1_ has shown promising effects, enhancing synaptic endocannabinoid availability through blockade of MAGL and FAAH has yielded mixed results. The pharmacological inhibition of MAGL – thus increasing synaptic availability of 2-AG – with JZL-184 showed the same reversal of acquisition latencies seen in 7-month 3xTg-AD mice as CB_1_ activation with WIN55212 [132], and improvements in spatial learning and memory with simultaneous repressed β-amyloid accumulation in 5xFAD mice [136]. However, unlike agonist treatment, JZL-184 had no improvement on functional M_2_/M_4_ mAChR coupling in 3xTg-AD mice [132]. Meanwhile, pharmacological inhibition of FAAH had little effect on cognitive impairment, plaque deposition or inflammatory markers in 5xFAD mice [135], indicating that the pharmacological enhancement of anandamide levels – in contrast to 2-AG enhancement – does little to improve symptoms of AD in this particular mouse model. On the other hand, FAAH-null 5xFAD mice showed a behavioural improvement in spatial memory tasks and diminished levels of both soluble amyloid and neuritic plaque density [135], indicating that perhaps a lifelong upregulation of anandamide availability is protective against the onset of AD-like symptoms in 5xFAD mice. Surprisingly, a paradoxical elevation of pro-inflammatory cytokines levels was also seen in these mice, and the attenuation of memory impairment was not reversed by administration of SR141716 – a CB_1_ antagonist – indicating that genetically elevated anandamide levels do not mediate improvements in spatial memory *via* CB_1_ [135]. Given that anandamide also activates GPR55 and CB_2_, it is possible that genetic deletion of FAAH leads to anandamide acting not only on CB_1_, but instead on the endocannabinoid system as a whole, to improve cognitive function and attenuate the onset of markers of AD. Indeed, as discussed above, the activation of GPR55 leads to the release of glutamate and thus supports LTP formation [111,112]. Furthermore, anandamide has been shown to bind to M_1_ and M_4_ mAChRs, where it is thought to bind to allosteric sites [137,138]. It is therefore possible that the CB_1_-independent properties of genetic FAAH ablation could, in part, be explained by activation of GPR55, CB_2_ or even binding to non-CB receptors such as the mAChRs.

As discussed above, upregulated CB_2_ receptor expression in the CNS has been recently implicated in the development of neuroinflammation, and as such could be a target for the treatment of AD. Indeed, genetic deletion of CB_2_ in 5xFAD mice (CB_2_R^−/−^ /5xFAD) showed a small but significant decrease in hippocampal neuritic plaque density [129]. However, these mice did not show any changes in Aβ1–42 levels or reduced microgliosis in comparison to their wildtype littermates [129]. Meanwhile, deletion of CB_2_ in APP/PS1 mice resulted in an improvement of cognitive and learning deficits with a concomitant reduction in neuronal loss, decreased plaque levels and increased expression of Aβ degrading enzymes [139]. In contrast, however, the pharmacological activation of the CB_2_ receptor *via* the CB_2_-selective agonist JWH-133 may also be beneficial in neurodegeneration. In an okadaic acid rat model of AD, neurodegeneration, neuroinflammation, Aβ accumulation and impairment of cognitive function were all prevented following daily administration of JWH-133 over a two-week period [140]. Reductions in pathological hallmarks of disease were accompanied by a reversal of spatial memory impairment and anxiety to control levels [140]. Therefore, the precise role of CB_2_ in neurodegenerative disease remains unclear, although these data collectively indicate that targeting the CB_2_ receptor could prevent or reverse the formation of neuritic plaques and, importantly, reverse memory impairments in AD.

## 6 Cannabinoids modulate the M1 mAChR in pilocarpine-induced seizure

In addition to its role in learning and memory, the M_1_ mAChR has long been implicated in the mediation of seizure onset. While genetic disruption of M_1_ ablates seizure activity in mice [141], both inhibition of AChE by organophosphorous (OP) compounds [142,143] and treatment with pilocarpine induce seizures in rodents [144]. The latter shows a phenotype similar to human temporal lobe epilepsy, and is thus a widely used model of this disease state [144]. These effects are long-lasting, with pilocarpine-treated animals developing spontaneous seizures a few weeks after compound administration. Consistent with the notion that the spontaneous seizures in this model is driven by mAChRs is the fact that pre-treatment with muscarinic antagonists prior to pilocarpine treatment can block the development of spontaneous seizures. However, muscarinic antagonist administration after pilocarpine treatment does not terminate seizure activity. Therefore, mAChRs are required for the development, but not necessarily the maintenance of pilocarpine-induced spontaneous seizures [144].

The effectiveness of directly targeting CB_1_ in seizure states can be illustrated by both CB_1_ knockout mouse models and CB_1_ antagonist administration, both of which increase pilocarpine seizure sensitivity in the pilocarpine-induced seizure model [144]. However, this is not restricted to this particular seizure model, as CB_1_ ablation and antagonist treatment both produce proconvulsive phenotypes in electroshock, spontaneous and kainic acid seizure models of epilepsy [[145], [146], [147], [148]]. On the other hand, both endocannabinoids and CB_1_ receptor agonist administration can attenuate seizures in all of these models. Collectively, this is indicative that constitutive CB_1_ activity *via* endocannabinoids modulates not only mAChR-driven seizure activity, but also the onset of seizures through other neurotransmitter systems, which is reflective of the fact that the endocannabinoid system is indeed a modulatory system for a breadth of neurotransmitters. A protective role for CB_1_ has been described in the OP-induced seizure model, whereby CB_1_ antagonism with AM251 significantly increased mortality in mice [149]. Given that OP-induced seizures are mediated by inhibition of AChE – and thus an augmentation of synaptic acetylcholine – this therefore indicates that presynaptic CB_1_ receptors play a crucial role in the attenuation of presynaptic acetylcholine release. However, the agonism of CB_1_ as an approach to the treatment of seizures must be approached with caution, as a maximal dentate activation model of limbic seizures in anaesthetised rats indicated that direct CB_1_ agonism with WIN55212 resulted in impairments of LTP in addition to seizure attenuation [150]. The same study found that FAAH inhibition with URB597 inhibited seizure in a CB_1_-dependent manner, while avoiding the deleterious effects seen with WIN55212 [150]. Therefore, while targeting CB_1_ in seizure models shows therapeutic promise, it may also result in adverse effects. Meanwhile, enhancing endocannabinoid tone appears to result in the same anticonvulsant improvements while avoiding deleterious effects of CB_1_ agonism. Of great importance, however, is that these studies highlight the involvement and protective nature of CB_1_ in the modulation of seizures driven by mAChR overstimulation, and the therapeutic potential for targeting the endocannabinoid system in disorders of cholinergic dysfunction.

Given that the World Health Organisation states that epilepsy continues to affect some 50 million individuals worldwide, the attenuation of seizures *via* both phyto- and synthetic cannabinoids highlights the enormous potential for further defining the endocannabinoid system as a target for seizures. Anecdotal reports arising from the recent growth of public interest in the use of cannabinoids particularly emphasise the effectiveness of CBD over THC. Indeed, recent placebo-controlled trials have shown that the daily addition of 20 mg per kg of bodyweight of CBD to antiepileptic medication in Dravet syndrome resulted in a reduction in convulsive-seizure of at least 50% in 43% of participants [151], while reducing the frequency of drop seizures in Lennox-Gastaut syndrome by 41.9% [152]. The effectiveness and popularity of CBD over may be reflected in the differing pharmacology whereby, as discussed earlier, THC is a partial agonist at CB_1_ and CB_2_, while CBD is a NAM at CB_1_, a partial agonist at CB_2_ and an inhibitor of anandamide uptake. Adding yet further complexity to the role of cannabinoids in seizure modulation, THC may also be a competitive inhibitor of AChE *in vitro* [153], thus implying that THC could actually enhance synaptic acetylcholine levels, which would be deleterious in ACh-driven seizures. Therefore, the somewhat complex pharmacological properties of CBD – including blockade of anandamide uptake – may combine to attribute anticonvulsant effects, while direct agonism at CB_1_ – as seen with THC – may be associated with more adverse effects. Therefore, while the phyto- and synthetic cannabinoids show great promise in the treatment and prevention of seizures, more research is required to elucidate the precise role of the endocannabinoid system, particularly with regards to its role in crosstalk with the muscarinic receptors in disease states, prior to the exploitation of cannabinoids for therapeutic benefit.

## 7 Concluding remarks

The endocannabinoid system is a relatively new research field, the true therapeutic potential of which has not yet been fully elucidated. While many efforts in AD research strive to directly target the mAChRs through either orthosteric or allosteric compounds, emerging evidence indicates that, as modulatory receptors, endocannabinoid receptor crosstalk with mAChRs may serve as a way to indirectly fine tune cholinergic dysregulation. This may be of particular importance during discrete disease stages, such as during the upregulation of CB_1_ in the early stages of Alzheimer's disease. However, targeting the endocannabinoid system is not without its challenges, as CB_1_ is the most abundantly expressed GPCR in the CNS and the principal regulator of neuronal function with diverse effects on neuronal responses [149]. Therefore, a deeper understanding around the specific roles of these receptors on discrete neuronal populations, and strategies for directly targeting cannabinoid receptors in areas of the CNS relevant to disease are required before the endocannabinoid can be fully exploited as a drug target. Meanwhile, public perception around the recreational use of cannabinoids and the harmful nature of prolonged cannabis-based drug abuse has hampered the commitment of the scientific community to test the notion of the endocannabinoid system as a legitimate target in certain disease settings. Collectively, this highlights the continued need for research to be aimed towards deepening our understanding of the endocannabinoid system to optimise drug design for these emerging targets, and their role in disorders of cholinergic dysfunction such as seizure induction and AD.
